# Group B* Streptococcus* Induces a Robust IFN-*γ* Response by CD4^+^ T Cells in an* In Vitro* and* In Vivo* Model

**DOI:** 10.1155/2016/5290604

**Published:** 2016-02-16

**Authors:** Damian Clarke, Corinne Letendre, Marie-Pier Lecours, Paul Lemire, Tristan Galbas, Jacques Thibodeau, Mariela Segura

**Affiliations:** ^1^Laboratory of Immunology, Faculty of Veterinary Medicine, University of Montreal, Saint-Hyacinthe, QC, Canada J2S 2M2; ^2^Laboratory of Molecular Immunology, Department of Microbiology, Infectiology and Immunology, University of Montreal, Montreal, QC, Canada H3C 3J7

## Abstract

Group B* Streptococcus* (GBS) serotype III causes life-threatening infections. Cytokines have emerged as important players for the control of disease, particularly IFN-*γ*. Although potential sources of this cytokine have been proposed, no specific cell line has ever been described as a leading contributor. In this study, CD4^+^ T cell activation profiles in response to GBS were evaluated through* in vivo*,* ex vivo,* and* in vitro* approaches. Total splenocytes readily produce a type 1 proinflammatory response by releasing IFN-*γ*, TNF-*α*, and IL-6 and actively recruit T cells via chemokines like CXCL9, CXCL10, and CCL3. Responding CD4^+^ T cells differentiate into Th1 cells producing large amounts of IFN-*γ*, TNF-*α*, and IL-2.* In vitro* studies using dendritic cell and CD4^+^ T cell cocultures infected with wild-type GBS or a nonencapsulated mutant suggested that GBS capsular polysaccharide, one of the major bacterial virulence factors, differentially modulates surface expression of CD69 and IFN-*γ* production. Overall, CD4^+^ T cells are important producers of IFN-*γ* and might thus influence the course of GBS infection through the expression balance of this cytokine.

## 1. Introduction

Group B* Streptococcus* (GBS) or* Streptococcus agalactiae* is the main cause of life-threatening infections in newborns worldwide [1, 2]. GBS also affects pregnant women, elders, and immunocompromised patients [3]. Type III GBS is frequently involved in neonatal infections and is the most common type in GBS meningitis [1, 2].

Cytokines are important for controlling GBS disease, although exaggerated responses might be dangerous [4, 5]. While IL-10, IL-12, and IL-18 are beneficial [6–9], TNF-*α* contributes to GBS-induced sepsis [7, 10]. IFN-*γ* appears promising for control of GBS disease; IL-12 and IL-18 exert therapeutic effects by stimulating immune cells to produce IFN-*γ* [6, 8, 9], IFN-*γ* production is impaired in neonates and this might partly explain their susceptibility to GBS infection [8, 11, 12], and IFN-*γ* inhibits GBS survival in human endothelial cells [13]. Although NK and NKT cells have been proposed to secrete IFN-*γ* in response to GBS [14, 15], no specific cell line has been clearly identified yet as a major source.

Activated CD4^+^ T cells can differentiate into T helper (Th) cell types depending on the signals they receive. Th1 cells readily produce IFN-*γ* upon activation. GBS-infected dendritic cells (DCs) produce large amounts of proinflammatory cytokines like TNF-*α*, IL-6, and IL-12 [16] that could activate T cells. Furthermore, GBS-activated DCs release chemokines recruiting T cells, like CXCL9 and CXCL10 [16]. Although these evidences support IFN-*γ* production by T cells [17, 18], the participation of CD4^+^ T cells during GBS-induced disease is unknown.

GBS possesses a thick sialylated polysaccharide capsule (CPS) [19]. It is known as the most important factor for GBS survival within the host and interferes with innate defense mechanisms [4, 20, 21]. Encapsulated GBS is highly internalized by DCs but survives better intracellularly than its nonencapsulated counterpart. Bacterial internalization and the presence of CPS are also related to modulation of several cytokines and chemokines released by GBS-infected DCs [16, 22, 23].

It is hypothesized here that GBS drives CD4^+^ T cells differentiation into IFN-*γ*-producing Th1 cells and that the CPS can modify this response. The role of CD4^+^ T cells in the immune response against GBS type III was investigated using* in vivo*,* ex vivo*, and* in vitro* approaches in a mouse model. A nonencapsulated GBS mutant was included to dissect the role of this virulence factor in T cell activation.

## 2. Materials and Methods

### 2.1. Bacterial Strains

COH-1, a highly encapsulated type III GBS isolate extensively described in [16, 22, 24], and its isogenic nonencapsulated (Δ*cpsE*) mutant [16, 22] were used. GBS strains were cultivated as described previously [22].

### 2.2. Antibodies

Anti-mouse antibodies (BioLegend unless otherwise noted) used for FACS analysis were as follows: FITC-conjugated anti-CD3 (17A2) and anti-CD4 (GK1.5; BD Pharmingen); PE-conjugated anti-CD4 (GK1.5), anti-CD19 (6D5), anti-CD69 (H1.2F3; BD Pharmingen), anti-IFN-*γ* (XMG1.2; eBioscience), anti-TNF-*α* (MP6-XT22; eBioscience), and anti-IL-2 (JES6-5H4; eBioscience); PE-Cy7-conjugated anti-NK-1.1 (PK136) and anti-CD44 (IM7; BD Pharmingen); APC-conjugated anti-IFN-*γ* (XMG1.2), anti-TNF-*α* (MP6-XT22) and anti-IL-7R*α* (A7R34), and BV421-conjugated anti-CD62L (MEL-14).

### 2.3. Mice and Experimental Infections

Five-week-old female C57BL/6 mice (Charles River Laboratories) were used for all experiments. The University of Montreal Animal Welfare Committee guidelines and policies were followed. On the day of the experiment, 0.5 mL of the bacterial suspension (10^6^, 10^7^, or 10^8^ CFU) or sterile vehicle solution was administrated intraperitoneally (i.p.). Mortality and clinical signs were monitored [25]. Blood samples (5 *μ*L) were collected at different times after infection. Bacteremia (number of CFU/mL) was determined by plating samples onto blood agar using an Automated Spiral Plater (Spiral Biotech).

### 2.4. Generation of Bone Marrow-Derived DCs and Isolation of Splenic CD4^+^ T Cells

DCs were generated as described previously from naïve mice [16]. Cell purity was 86–90% CD11c^high^ and F4/80^−/dim^ cells by FACS analysis as reported previously [16]. For purification of untouched CD4^+^ T cells, spleens (from either naïve or infected mice) were harvested, perfused with RPMI complete medium (Gibco), and pressed gently through a sterile fine wire mesh. After red blood cells lysis (eBioscience), total splenocytes were suspended in 2 mM EDTA-PBS solution and separated using Lympholyte-M density gradient (Cedarlane Lab.). Low-density cells at the interphase were purified by magnetic-activated cell sorting (MACS) negative selection (Miltenyi Biotec). The enriched CD4^+^ T cells had >96% purity by FACS analysis using CD3 and CD4 antibodies (data not shown). For all experiments, cells were incubated at 37°C, 5% CO_2_.

### 2.5. *In Vivo* Infection Model

**Figure 1:**
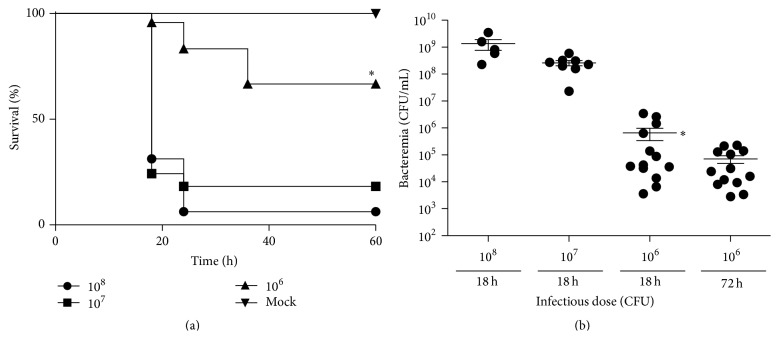
Survival curves and bacteremia levels of GBS-infected C57BL/6 mice. (a) Mice (*n* = 16) were injected intraperitoneally with different doses of wild-type GBS serotype III strain COH-1 and survival levels recorded. Mock-infected animals (injected with the vehicle solution) were used as controls. (b) Systemic bacteremia levels of infected mice were monitored at 18 h after infection (for mice infected with 10^6^, 10^7^, and 10^8^ CFU) and at 72 h after infection (for mice infected with 10^6^ CFU). Blood was drawn by tail puncture and serially diluted in PBS prior to plating on blood agar dishes. Individual colonies were counted and data expressed as CFU/mL of blood. ^*∗*^ *P* < 0.05, compared to higher infectious doses.

For survival curves and selection of the infectious dose, mice (*n* = 16) were injected i.p. with 10^6^, 10^7^, or 10^8^ CFU (strain COH-1) and clinical signs were monitored. Based on the obtained data (Figure 1(a)), mice were injected i.p. with 10^6^ CFU. Surviving animals who displayed clinical signs were boosted with 10^6^ CFU 2 weeks after initial infection. Bacteremia was monitored during 72 h after primary infection or at 24 h after boost. Spleens of animals with clinical signs and positive bacteremia were harvested 96 h after primary infection or 48 h after boost (*n* = 2 per group × 5 individual experiments). Five hours before spleen collection, mice were injected i.p. with 200 *μ*g of Brefeldin A (eBioscience), a protein transport inhibitor. Control (mock-infected) animals were similarly treated. Brefeldin A was kept throughout the purification steps. The selected time points are based on pretrials analysis (data not shown). Purified CD4^+^ T cells were analyzed for cytokine production by intracellular flow cytometry (IC-FACS). Total splenocytes were analyzed for memory surface markers by multiparametric FACS. Cells were gated on CD3^+^ CD4^+^ double-positive cells, followed by gating CD44^high^ CD62L^−^ (effector [memory] T cells) and CD44^high^ CD62L^+^ (central memory T cells). Analysis with a fifth surface marker, IL-7R*α* ^+^, was used to further identify memory cells (CD44^high^ IL-7R*α* ^+^) within these two subsets [26, 27].

### 2.6. *Ex Vivo* Analysis of Total Splenocytes

Mice were injected i.p with 10^7^ CFU (strain COH-1) (*n* = 3 per group × 3 individual experiments). Spleens were harvested 6 h after infection. Total splenocytes (5 × 10^6^ cells/mL) were plated in complete medium without antibiotics and incubated for 48 and 72 h. After an initial 4 h incubation, the bacteriostatic agent chloramphenicol (12 *μ*g/mL, Sigma-Aldrich) was added to control the bacterial load as reported previously [16]. Total splenocytes from control (mock-infected) animals were similarly treated. Concanavalin A (ConA, 0.1 *μ*g/mL, Sigma-Aldrich) served as positive control. Supernatants were harvested at different time points for cytokine analysis. In selected experiments, Brefeldin A (3 *μ*g/mL) was added for the last 5 h of incubation, and total splenocytes or CD4^+^ T cells (MACS-isolated from the culture wells) were analyzed by IC-FACS after a total 48 h incubation. The culture conditions were selected based on pretrials (data not shown).

### 2.7. *In Vitro* DC-T Cell Coculture Model

DCs were plated in 48-well flat-bottom plates (10^5^ cells/well; 1 h) prior to a 1 h infection with COH-1 or Δ*cpsE* strains (MOI:1). After a 1 h treatment with 100 *μ*g/mL gentamycin and 5 *μ*g/mL penicillin G (Sigma-Aldrich) to kill extracellular bacteria as described previously [16], DCs were washed. Freshly isolated CD4^+^ T cells from naïve mice were added (5 : 1 T cell/DC ratio; 8 and 24 h). Cocultures incubated with medium alone or ConA (0.1 *μ*g/mL) served as negative and positive controls, respectively. Cells were harvested for FACS analysis of surface marker expression. For T cell cytokine expression, after a 48 h incubation, plates were centrifuged and replenished with fresh medium containing 10 ng/mL of mouse rIL-2 (Miltenyi Biotec). After a 3-day resting period, T cells were harvested, washed, and seeded into 96-well flat-bottom culture plates coated with 5 *μ*g/mL of anti-mouse-CD3 mAb (BD Pharmingen) (10^5^ cells/well; 48 h). Supernatants were harvested for ELISA testing. Single cell cultures (DCs or T cells alone) served as controls.

### 2.8. Cytokine and Chemokine Quantification by ELISA

Levels of IL-6, IL-10, IFN-*γ*, TNF-*α*, CCL3, CXCL9, and CXCL10 in cell culture supernatants were measured by sandwich ELISA using pair-matched antibodies (R&D Systems or eBioscience). Sample dilutions giving OD readings in the linear portion of a standard curve were used to quantify the levels of each cytokine. The results include at least three independent ELISA measurements.

### 2.9. FACS Analysis

For multiparametric IC-FACS, total splenocytes were treated with FcR-blocking reagent (Fc*γ*III/II Rc Ab; BD Pharmingen) for 15 min on ice. Cells were stained for CD19, NK-1.1, CD3, and/or CD69 (30 min on ice), fixed, and permeabilized (eBioscience). After intracellular staining for IFN-*γ* or TNF-*α* (45 min, room temperature), FACS was performed using a FACSCanto II instrument (BD Biosciences). Fluorescence Minus One (FMO) control staining was performed for proper analysis and gating of target cells. For IC-FACS of MACS-purified CD4^+^ T cells from* in vivo* or* ex vivo* experiments, cells were stained intracellularly for IFN-*γ*, TNF-*α*, and IL-2 as described above and analyzed with a FACSCalibur instrument (BD Biosciences). For analysis of the memory response by multiparametric FACS, total splenocytes were blocked and surface-stained for CD3, CD4, CD44, CD62L, and IL-7R*α* (45 min on ice). FACS was performed using a FACSCanto II instrument. Cells from* in vitro* cocultures were surface-stained for CD4 and CD69 (30 min on ice). FACS was performed using a Cell Lab Quanta*™* SC MPL MultiPlate Loader instrument (Beckman Coulter).

### 2.10. Statistical Analysis

Survival curves of infected mice were generated using Kaplan-Meier plots and log-rank (Mantel-Cox) tests allowed comparison between groups. Bacteremia levels were compared using the Mann-Whitney test. Cytokine data (expressed as means ± SEM) and FACS data were analyzed for significance using Student's unpaired *t*-test. All analyses were performed using the Sigma Plot System (Systat Software). A *P* < 0.05 was considered as statistically significant.

## 3. Results

### 3.1. Survival of GBS-Infected Mice Is Dose-Dependent

After 18 h, infection with 10^7^ or 10^8^ CFU of COH-1 strain resulted in 75% and 69% mortality (*P* > 0.05), respectively (Figure 1(a)). Mortality continued to increase until 24 h after infection to 82% and 94% (*P* > 0.05), respectively, and was maintained until 60 h after infection when the experiment was terminated. Mice infected with 10^6^ CFU were significantly less prone to mortality than mice from the other groups. At 18 h after infection, only a 6% mortality rate was observed, which was significantly lower than in the other groups (*P* < 0.05). Mortality continued to increase at 24 and 36 h after infection yet remained significantly lower than in mice infected with higher doses (*P* < 0.05). Indeed, mice infected with 10^7^ or 10^8^ CFU manifested intense clinical signs as early as 8 h after infection, while 10^6^ CFU usually induced less severe signs starting 12 h after infection.

Bacteremia induced by COH-1 infection was consistent with survival curves (Figure 1(b)). Mice infected with 10^7^ or 10^8^ CFU showed high bacteremia at 18 h after infection and reached an average of 2.6 × 10^8^ and 1.3 × 10^9^ CFU/mL, respectively. In contrast, mice infected with 10^6^ CFU showed significantly lower bacteremia and reached an average of 5.7 × 10^5^ CFU/mL. High mortality rates impeded follow-up of bacteremia in mice infected with high doses. However, in mice infected with 10^6^ CFU, bacteremia slowly decreased, reaching an average of 7.2 × 10^4^ CFU/mL at 72 h after infection.

### 3.2. Splenocytes Produce Type-1 Proinflammatory Cytokines in Response to Encapsulated GBS Infection

**Figure 2:**
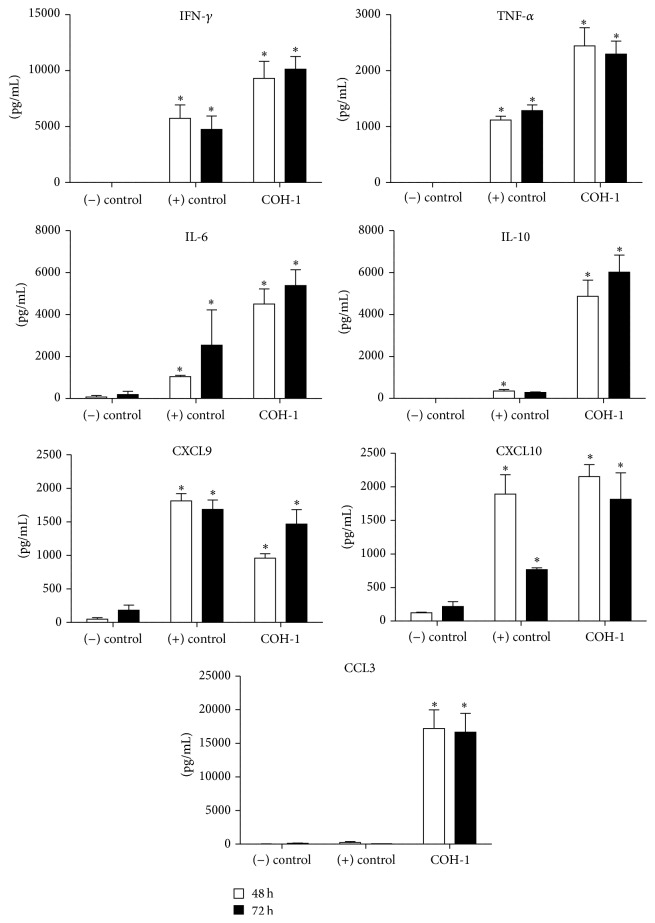
*Ex vivo* cytokine and chemokine production profile by total splenocytes. C57BL/6 mice were injected intraperitoneally with a dose of 10^7^ CFU of wild-type GBS serotype III strain COH-1 (*n* = 3 per group × 3 individual experimental infections). Spleens were harvested 6 h after infection and total splenocytes plated at 5 × 10^6^ cells/well. After 4 h of incubation, the bacteriostatic agent chloramphenicol (12 *μ*g/mL) was added to the culture to prevent cell toxicity. Cells were then incubated for 48 h and 72 h and supernatants were collected for cytokine analysis by ELISA. Nonstimulated cells from mock-infected animals served as negative (−) control for basal expression. Cells stimulated with Concanavalin A (0.1 *μ*g/mL) were used as positive (+) control. Data are expressed as means ± SEM (in pg/mL) from 3 different experimental infections. ^*∗*^ *P* < 0.05 indicates statistically significant difference compared to (−) control cells.

Before investigating T cell activation, the splenic immunological environment was characterized. Total splenocytes from mice infected with COH-1 strain were incubated* ex vivo* for 48 and 72 h (Figure 2). High amounts of IFN-*γ*, TNF-*α*, and IL-6 were detected (*P* < 0.05), suggesting a type-1 proinflammatory response. IL-10 was also upregulated in infected spleens, suggesting a homeostatic role. Important chemokines for T cell recruitment were also detected: CXCL9, CXCL10, and CCL3 (*P* < 0.05). It is worth noting that CXCL9 and CXCL10 are mainly released in response to IFN-*γ* activation [28], thus in agreement with the observed high levels of IFN-*γ* produced by GBS-infected splenocytes. No significant differences were observed between 48 and 72 h cultures, except for CXCL9 where maximal production was delayed to 72 h of incubation.

### 3.3. Activated CD4^+^ T Cells Contribute to IFN-*γ* Production during Encapsulated GBS Infection

**Figure 3:**
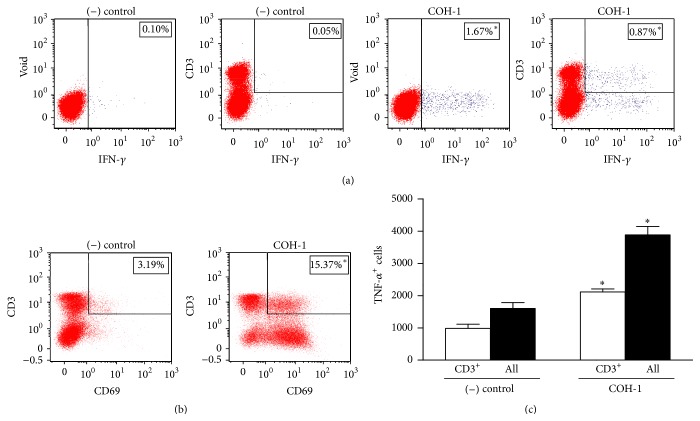
*Ex vivo* analyses of cellular sources of IFN-*γ* during GBS infection. C57BL/6 mice were injected intraperitoneally with a dose of 10^7^ CFU of wild-type GBS serotype III strain COH-1 (*n* = 3 per group × 3 individual experimental infections). Spleens were harvested 6 h after infection and total splenocytes plated at 5 × 10^6^ cells/well. After 4 h of incubation, the bacteriostatic agent chloramphenicol (12 *μ*g/mL) was added to the culture to prevent cell toxicity. Nonstimulated cells from mock-infected animals served as negative (−) control for basal expression. Total splenocytes were incubated for 48 h with Brefeldin A (3 *μ*g/mL) added during the last 5 h of incubation. Cells were harvested and intracellularly stained for IFN-*γ* (a) or surface-stained for CD69 (b) in combination with several surface markers for multiparametric FACS analysis. Representative data from 3 different experimental infections based on CD3^+^ population or total splenic population (Void). (c) Number of TNF-*α* ^+^ cells within the CD3^+^ population or total splenic population (All). Data are expressed as means ± SEM from 3 different experimental infections; ^*∗*^ *P* < 0.05 indicates statistically significant difference compared to (−) control cells. Fifty thousand gated events were acquired per sample and data analysis was performed using FACSDiva*™* software.

With current understanding of the splenic environment, the contribution of activated T cells to cytokine production was investigated. A multiparametric IC-FACS analysis of IFN-*γ* production from* ex vivo* total splenocytes cultures was performed. CD3^+^ T cells markedly contributed to the IFN-*γ* response in the spleen of infected mice (Figure 3(a); *P* < 0.05). NKT cells (NK1.1^+^ CD3^+^) produced very low levels of IFN-*γ* (data not shown). NK cells (NK1.1^+^) were major contributors to IFN-*γ* production within the CD3^−^ population (data not shown). As expected, B cells (CD19^+^) did not produce significant levels of this cytokine (data not shown). Activated CD3^+^ T cells also contributed to approximately half the production of TNF-*α* by splenic cells (Figure 3(c); *P* < 0.05). Compared to control mice, splenocytes from infected animals showed a significant increase in surface expression of the early activation marker CD69. High expression of CD69 was also observed within the CD3^+^ population (Figure 3(b); *P* < 0.05).

**Figure 4:**
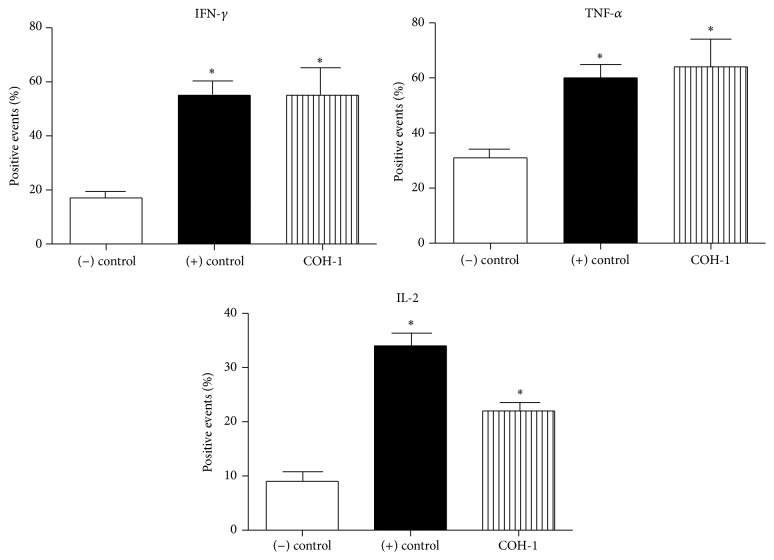
*Ex vivo* analyses of CD4^+^ T cell contribution to cytokine production. C57BL/6 mice were injected intraperitoneally with a dose of 10^7^ CFU of wild-type GBS serotype III strain COH-1 (*n* = 3 per group × 3 individual experimental infections). Spleens were harvested 6 h after infection and total splenocytes plated at 5 × 10^6^ cells/well. After 4 h of incubation, the bacteriostatic agent chloramphenicol (12 *μ*g/mL) was added to the culture to prevent cell toxicity. Nonstimulated cells from mock-infected animals served as negative (−) control for basal expression. Cells stimulated with Concanavalin A (0.1 *μ*g/mL) were used as positive (+) control. Total splenocytes were incubated for 48 h. Brefeldin A (3 *μ*g/mL) was added during the last 5 h of incubation and CD4^+^ T cells were MACS-isolated from the culture, stained intracellularly for different cytokines, and analyzed by FACS. Data are expressed as means ± SEM (in % of positive cells) from 3 individual experimental infections. ^*∗*^ *P* < 0.05 indicates statistically significant difference compared to (−) control cells. Twenty thousand gated events were acquired per sample and data analysis was performed using CellQuest software. Histograms were drawn based on PE-control stain and were plotted on logarithmic scales.

**Figure 5:**
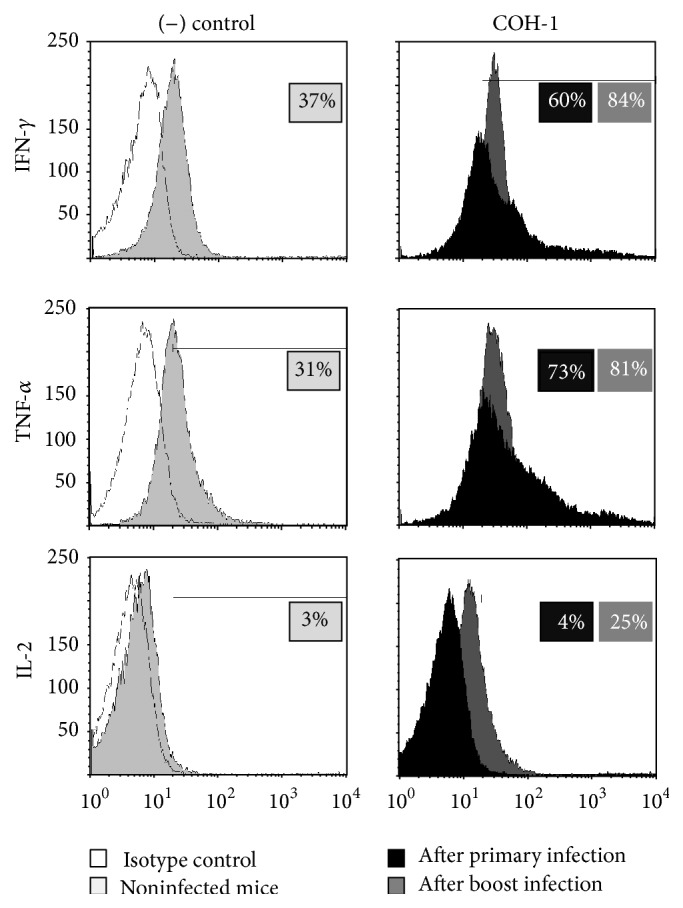
*In vivo* CD4^+^ T cell contribution to cytokine production during primary and secondary GBS infections. C57BL/6 mice were injected intraperitoneally with a dose of 10^6^ CFU of wild-type GBS serotype III strain COH-1. Surviving animals who had previously displayed clinical signs were boosted with a second dose of 10^6^ CFU of GBS strain COH-1 two weeks after initial infection. Spleens of animals with clinical signs and positive bacteremia were harvested 96 h after primary infection or 48 h after boost infection (*n* = 2 per group × 5 individual experimental infections). Five hours prior to spleen collection, mice were injected with Brefeldin A (200 *μ*g). (−) Control animals were similarly treated. Splenic CD4^+^ T cells were MACS-purified, stained intracellularly for different cytokines, and analyzed by FACS. Representative data from 5 different experimental infections. Cytokine basal expression levels in (−) control animals were similar at 96 h after primary mock-infection and 48 h after secondary mock-infection. Representative histograms from the latter time point were selected for the figure. Twenty thousand gated events were acquired per sample and data analysis was performed using CellQuest software. Histograms were drawn based on PE-control stain and were plotted on logarithmic scales. It should be noted that isotype controls are the same in both groups, but only displayed on left panels to simplify the figure.

**Figure 6:**
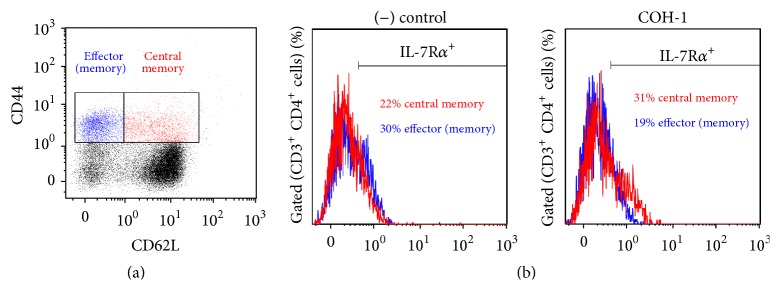
*In vivo* generation of memory CD4^+^ T cells during GBS infection. C57BL/6 mice were injected intraperitoneally with a dose of 10^6^ CFU of wild-type GBS serotype III strain COH-1. Surviving animals who had previously displayed clinical signs were boosted with a second dose of 10^6^ CFU of GBS strain COH-1 two weeks after initial infection. Spleens of animals with clinical signs and positive bacteremia were harvested 48 h after boost infection. Total splenocytes were stained and analyzed by multiparametric FACS. (a) Cells were gated on CD3^+^ CD4^+^ double-positive cells, followed by gating CD44^high^ CD62L^−^ (effector [memory] T cells) and CD44^high^ CD62L^+^ (central memory T cells). A histogram from a representative control (mock-infected) mouse was selected for the figure. (b) A fifth surface marker, IL-7R*α* ^+^, was used to further identify memory cells (CD44^high^ IL-7R*α* ^+^) within the CD44^high^ CD62L^−^ (effector [memory] T cells) and CD44^high^ CD62L^+^ (central memory T cells). IL-7R*α* ^+^ cells reflect memory cells within these respective populations. Histograms from representative control (mock-infected) and infected mice were selected for the figure. Thirty thousand events gated on CD3^+^ CD4^+^ cells were acquired per sample and data analysis was performed using Kaluza® Flow Analysis software.

CD4^+^ T cells were isolated from* ex vivo* total splenocyte cultures and analyzed by IC-FACS to specifically evaluate their role (Figure 4). Activated CD4^+^ T cells contributed to the production of IFN-*γ* and TNF-*α*. Low levels of intracellular IL-2 were also observed (Figure 4).* In vivo* experiments confirmed these results; CD4^+^ T cells directly isolated from the spleen of infected mice 96 h after primary infection showed that they contribute to the production of IFN-*γ* and TNF-*α*. Intracellular levels of IL-2 were hardly detected during a primary infection (Figure 5, black histograms). CD4^+^ T cells isolated 48 h after boost displayed an enhanced contribution to IFN-*γ*, TNF-*α*, and IL-2 production (Figure 5, dark grey histograms). This is consistent with the generation of memory CD4^+^ T cells (CD44^high^ IL-7R*α* ^+^) observed at that time with the increase in IL-7R*α* ^+^ cells in the central memory subset (CD44^high^ CD62L^+^) (Figures 6(a) and 6(b), red population and histograms). The decrease in IL-7R*α* ^+^ cells in the effector (memory) subset (CD44^high^ CD62L^−^) likely reflects cellular migration from the spleen to peripheral tissues (Figures 6(a) and 6(b), blue population and histograms).

### 3.4. The CPS of GBS Modulates Cytokine Release by CD4^+^ T Cells

**Figure 7:**
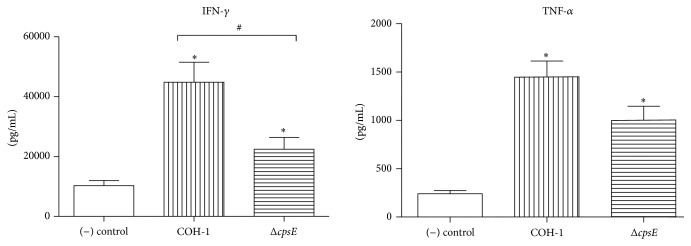
Role of bacterial capsular polysaccharide in the modulation of cytokine production by CD4^+^ T cells. Dendritic cells (DCs) were infected with either wild-type GBS strain COH-1 or its nonencapsulated isogenic mutant Δ*cpsE* (MOI:1) for 1 h. Extracellular bacteria were killed by antibiotic treatment and cultures washed prior to addition of freshly isolated splenic CD4^+^ T cells from naïve mice (T cell : DC ratio of 5 : 1). Cocultures were incubated for 48 h, resuspended in fresh medium containing 10 ng/mL of IL-2 for 72 h (resting period), and then transferred to anti-CD3 coated plates for 48 h. Supernatants were then collected and cytokines quantified by ELISA. Nonstimulated cocultures served as negative (−) controls for basal expression. Data are expressed as means ± SEM (in pg/mL) from 5 different experiments. ^*∗*^ *P* < 0.05 indicates statistically significant differences compared to (−) control. ^#^ *P* < 0.05 indicates statistically significant differences between cocultures infected with wild-type strain COH-1 and those infected with the nonencapsulated mutant Δ*cpsE*.

As GBS is a well-encapsulated bacterium, the impact of CPS on CD4^+^ T cell activation was evaluated by comparing COH-1 with its nonencapsulated mutant, Δ*cpsE*, in an* in vitro* DC-T cell coculture system. Since nonencapsulated GBS mutants are rapidly cleared from circulation [20],* in vivo* comparison was impossible. Coculture supernatants were tested by ELISA for CD4^+^ T cell-derived cytokines. No significant cytokine production was observed in single cell cultures (DCs or T cells alone) that served as controls (data not shown). COH-1-activated cocultures showed extremely high levels of IFN-*γ* (~45000 pg/mL) and significant levels of TNF-*α* (~1500 pg/mL). Δ*cpsE*-activated cocultures showed a significant reduction in IFN-*γ* production (Figure 7; *P* = 0.012), along with reduced TNF-*α* production, although this difference was not statistically significant (*P* = 0.053). Overall, these results suggest that nonencapsulated GBS-pulsed DCs induce reduced cytokine production by CD4^+^ T cells compared to encapsulated GBS-pulsed DCs.

### 3.5. The CPS of GBS Affects Surface Expression of CD69 on Activated CD4^+^ T Cells

**Figure 8:**
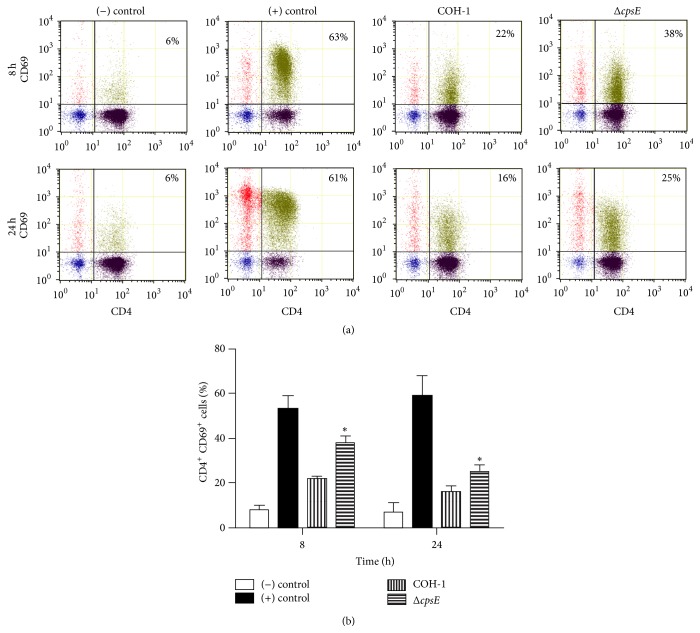
Role of bacterial capsular polysaccharide in the modulation of CD4^+^ T cell surface expression of CD69. Dendritic cells (DCs) were infected with either wild-type GBS strain COH-1 or its nonencapsulated isogenic mutant Δ*cpsE* (MOI:1) for 1 h. Extracellular bacteria were killed by antibiotic treatment and cultures washed prior to addition of freshly isolated splenic CD4^+^ T cells from naïve mice (T cell : DC ratio of 5 : 1). Cocultures were incubated for 8 h and 24 h, cells were harvested, and CD69 expression was analyzed by FACS. Cocultures incubated with medium alone or Concanavalin (0.1 *μ*g/mL) served as negative (−) and positive controls (+), respectively. (a) Representative data from 3 different experiments. Twenty thousand gated events were acquired per sample and data analysis was performed using Cell Lab Quanta Collection/Analysis software. Quadrants were drawn based on FITC- and PE-control stains and were plotted on logarithmic scales. Numbers in the upper quadrants indicate the % of CD4^+^ CD69^+^ cells. (b) Data are expressed as means ± SEM from 3 different experimental infections; ^*∗*^ *P* < 0.05 indicates statistically significant differences between cocultures infected with wild-type strain COH-1 and those infected with the nonencapsulated mutant Δ*cpsE*.

In addition to cytokine production, expression of surface molecules is essential for proper T cell activation. The effect of CPS on CD69 expression on activated CD4^+^ T cells was investigated. In COH-1-activated cocultures, CD69 expression on CD4^+^ T cells was significantly lower than in Δ*cpsE*-activated cocultures after an 8 h incubation (Figure 8; *P* < 0.01). CD69 expression remained lower in COH-1-activated cocultures at 24 h (*P* < 0.05), although the difference and levels of expression were less pronounced. After 48 h, no significant differences in CD69 expression were observed between strains (data not shown).

## 4. Discussion

Although interactions between GBS and innate immune cells are increasingly documented, activation profiles of adaptive immune cells have never been investigated. This is the first study evaluating CD4^+^ T cells contribution to GBS immune response using* in vivo*,* ex vivo*, and* in vitro* analysis.

While cytokines contribute to host defense development, they can also exacerbate GBS-induced pathologies. Initial* ex vivo* analysis of cytokine production by total splenocytes from encapsulated GBS-infected mice revealed the presence of IFN-*γ*, TNF-*α*, IL-6, and IL-10. Production of IFN-*γ*, TNF-*α*, and IL-6 suggests a type-1 proinflammatory response being developed shortly after infection, while IL-10 production can be related to immune regulation. Interestingly, TNF-*α* and IL-6 have routinely been reported as mediators of GBS sepsis [7, 10]. This result might also highlight the homeostatic role of IL-10. Indeed, IL-10 was shown to reduce TNF-*α* and thus protect neonatal mice from developing GBS sepsis [7].

DCs, monocytes, and macrophages are known to secrete TNF-*α*, IL-6, and/or IL-10 when responding to GBS [16, 17, 29–31]. However, sources of IFN-*γ* remain poorly identified. Early works reporting IFN-*γ* production used GBS-infected total splenocytes or mixed mononuclear cells, without identifying the cellular source [8–10, 17]. The present study defined the role of T cells in IFN-*γ* production.* Ex vivo* and* in vivo* analysis showed that CD4^+^ T cells are important producers of IFN-*γ* and TNF-*α* during GBS infection. Activated CD4^+^ T cells also produce low, but still significant levels of IL-2, suggesting the development of a Th1 response. CD4^+^ T cells produce the same pattern of cytokines more efficiently after a boost infection, likely thanks to the memory response [32]. An important contribution of NK cells to the IFN-*γ* response was also evidenced* in vivo*, in accordance with previous* in vitro* studies with splenocytes from severe combined immunodeficiency mice [15]. IFN-*γ* production by NKT cells was very limited during GBS infection, even at earlier time points (unpublished observations), although purified GBS glycolipids have been shown to activate NKT cells [14].

Early chemokine release by innate immune cells attracts T cells to the site of infection.* Ex vivo* analysis of chemokine production by total splenocytes suggested that T cells are actively recruited via CCL3, CXCL9, and CXCL10. Interestingly, CXCL9 and CXCL10 are two CXCR3 ligands, both induced by IFN-*γ*. CXCR3 is rapidly upregulated on naive T cells following activation and remains preferentially highly expressed on Th1 cells [28]. Different splenic cell types, like DCs, might produce these chemokines in response to GBS [16]. Although upregulation of* Cxcl10* gene expression was observed in mouse peritoneal macrophages [31], GBS was unable to induce either CXCL10 or CXCL9 secretion by these cells [33]. Nevertheless, both macrophages and DCs seem to contribute to CCL3 production [31, 33, 34].

As GBS possesses a thick CPS, its most important virulence factor, the potential of CPS to modulate CD4^+^ T cell activation was investigated. Similarly to* ex vivo* and* in vivo* results, DCs pulsed* in vitro* with encapsulated GBS induced the release of high levels of IFN-*γ* and TNF-*α* by CD4^+^ T cells. The production of IFN-*γ* was significantly decreased with nonencapsulated GBS. Production of TNF-*α* was also reduced. It is surprising that the loss of capsule does not trigger an exaggerated response or increased IFN-*γ* production by T cells, as reported for other encapsulated pathogens [35–37]. However, studies on GBS-activated DCs have shown similar trends; encapsulated GBS induced similar or stronger cytokine production by infected DCs than nonencapsulated GBS-infected counterparts [16, 34]. The only exception was IL-10, where production was significantly higher in DCs infected with the nonencapsulated mutant [16]. Two interrelated hypotheses were suggested to explain these observations: (a) increased IL-10 production by DCs reduces the production of other cytokines; or (b) more efficient killing of the nonencapsulated mutant reduces cytokine production by DCs [16, 22]. Moreover, it was reported that the presence of CPS modulates the endocytic pathways used by DCs for GBS uptake [22]. Since the route of entry influences the repertoires of epitopes presented to CD4^+^ T cells, the ensuing immune response might be affected [38]. Thus, in our DC-T cell coculture system, DC modulation by the nonencapsulated strain may lead to lower levels of IFN-*γ* production by CD4^+^ T cells.

In contrast to cytokine production, the surface expression of CD69 was higher (early time points) or similar (late time points) in CD4^+^ T cells cocultured with nonencapsulated mutant-pulsed DCs compared to encapsulated GBS-infected cocultures. However, this could just be related to different kinetics of CD69 expression. In fact, attempting to explain modulation of CD69 expression on CD4^+^ T cells is quite difficult, due to limited information on this marker. Indeed, characterization of its ligand has just started [39]. CD69 is known to be one of the earliest markers induced upon activation of T cells and acts as a signal-transmitting receptor for immunoregulatory events [40]. Of the few studies available on CD69 expression by T cells upon streptococcal infection, Harimaya et al. demonstrated a dose-dependent upregulation of CD69 on CD3^+^ T cells from peripheral blood lymphocytes infected with* Streptococcus pneumoniae*. Yet, authors failed to correlate CD69 expression and IFN-*γ* production by these target cells [41]. More recently, in a* S. pneumoniae* mouse model of infection, CD4^+^ T cells exhibited significant upregulation of CD69 in the spleen. As this response was MHC-II unrestricted, authors suggested that this increased CD69 expression on T cells might be due to secondary factors like cytokine release by other cells [42]. Likely, a polyclonal (indirect) activation of T cells in our system cannot be ruled out, although GBS failed to directly activate T cells without antigen-presenting cells (data not shown), similarly to that reported for* S. pneumoniae* [37, 42]. Finally, it has been suggested that CD69 plays an immunoregulatory role by preventing infection-induced immunopathology [43]. Enhanced expression of CD69 may result in reduced IFN-*γ* production by CD4^+^ T cells [44].

## 5. Conclusion

Undoubtedly, IFN-*γ* production by CD4^+^ T cells during GBS infection is crucial for host defense [8] but might also result in disease pathology, as suggested in the mouse model of pneumococcal sepsis [42]. Although this study characterized for the first time IFN-*γ* production by CD4^+^ T cells, a definitive understanding of all mechanisms regulating IFN-*γ* production during GBS infection requires further research. For instance, as the CPS confers a survival advantage to GBS [16, 22], persistence of GBS within antigen-presenting cells may affect their activation and thus the ensuing T cell immune response, including altered IFN-*γ* and CD69 expression balance early during infection.
